# Identification and X-ray Co-crystal Structure of a Small-Molecule Activator of LFA-1-ICAM-1 Binding**

**DOI:** 10.1002/anie.201310240

**Published:** 2014-04-01

**Authors:** Martin Hintersteiner, Jörg Kallen, Mario Schmied, Christine Graf, Thomas Jung, Gemma Mudd, Steven Shave, Hubert Gstach, Manfred Auer

**Affiliations:** The University of Edinburgh, School of Biological Sciences (CSE) and School of Biomedical Sciences (CMVM)CH Waddington Building, 3.07, The King's Buildings, Mayfield Road, Edinburgh, EH9 3JD (UK); Bioseutica BVCorso Elvezia 4, 6900 Lugano (Switzerland); Institute of Medical Chemistry, Medical Univ. of ViennaWaehringerstrasse 10, 1090 Vienna (Austria); Affiliation when work was performed: Novartis Institutes for BioMedical ResearchBrunnerstrasse 59, 1235 Vienna (Austria); Novartis Institutes for BioMedical ResearchNovartis Campus, 4056 Basel (Switzerland)

**Keywords:** high-throughput screening, LFA-1, OBOC libraries, small-molecule activators, structural biology

## Abstract

Stabilization of protein–protein interactions by small molecules is a concept with few examples reported to date. Herein we describe the identification and X-ray co-crystal structure determination of IBE-667, an ICAM-1 binding enhancer for LFA-1. IBE-667 was designed based on the SAR information obtained from an on-bead screen of tagged one-bead one-compound combinatorial libraries by confocal nanoscanning and bead picking (CONA). Cellular assays demonstrate the activity of IBE-667 in promoting the binding of LFA-1 on activated immune cells to ICAM-1.

The integrin leucocyte function-associated antigen 1 (LFA-1) is a heterodimeric immune receptor ubiquitously expressed on leucocytes. Its interaction with intercellular adhesion molecule 1 (ICAM-1) provides a critical recognition event between T-cells and antigen presenting cells in efforts by the immune system to pull off an early-stage cell-mediated immune response.^1^–^3^ The LFA-1/ICAM-1 axis has thus been explored as a target interaction for drug discovery.^4^–^7^ The conformational switch between low-affinity and high-affinity states of LFA-1 upon activation and ICAM-1 binding provides a challenge for the design of LFA-1–ICAM-1 interaction inhibitors.^8^, ^9^

While protein–protein interaction (PPI) inhibition by small molecules was long considered to be the ultimate art in drug design, even fewer examples of true agonists of PPIs have been reported.^10^–^13^ Conceptually, however, there is evidence for clear advantages of PPI stabilizers.^14^

LFA-1 activators would be useful for treatment of rare hereditary genetic disorders known as leucocyte adhesion deficiency (LAD) or as potential enhancers of tumor immunotherapy.^15^, ^16^ One apparent activator has been described.^17^ However, closer investigation revealed that the compound ultimately worked as an inhibitor by binding to the β2 MIDAS domain and by blocking leucocyte transendothelial migration.

One of the features of on-bead screening as an affinity based screening method is that the identified ligands can have different biological activity profiles and modes of action. The ability to confirm the primary on-bead hits by measuring their binding to target protein in homogenous solution is essential for an efficient use of one-bead one-compound (OBOC) library screening.^19^, ^20^ We have previously described two different methods of how to use tagged OBOC libraries to link on-bead screening with solution-based assays. PS/PS relies on including a generic tagging site for post-screening in situ labeling of hit compounds with a fluorescent dye and subsequent miniaturized affinity determination in solution.^21^, ^22^ The second approach uses a chemically stable UV-fluorophore, AIDA, as a permanent tag introduced on each compound during library synthesis.^23^, ^24^ The indazole dye, AIDA, is then used as mass-tag for decoding and as a tracer for affinity determination.

In an effort to apply this AIDA technology for the identification of LFA-1 ligands, we designed a target-biased diazepanone library (Scheme 1). This library, comprising a total of more than 75 000 compounds, was synthesized on 90 μm TentaGel beads using standard solid-phase synthesis methods.

**Scheme 1:**
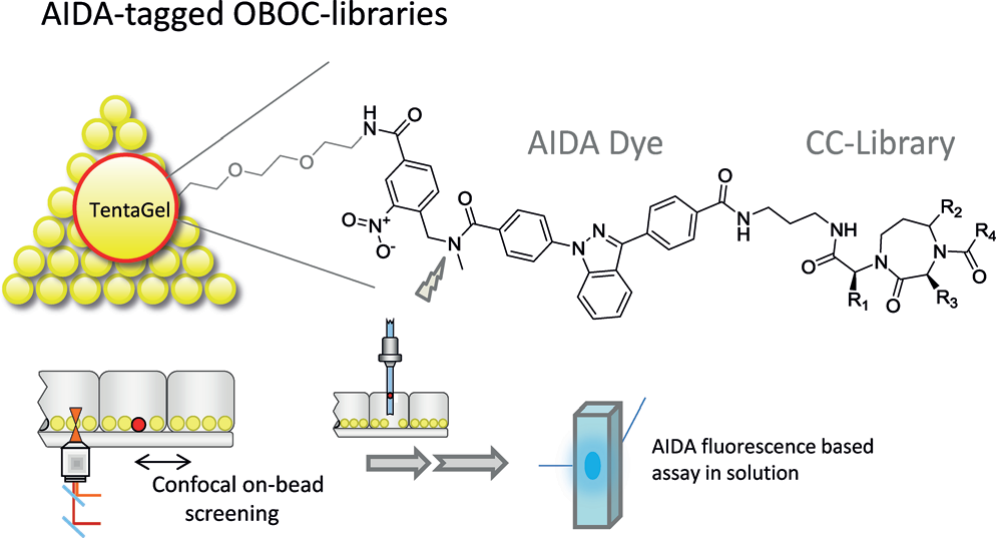
The AIDA-tagged one-bead one-compound library screening concept. The 1,3-diaryl-substituted indazole dye AIDA is incorporated into the solid-phase combinatorial library synthesis as first building block. A diaminopropane spacer separates the combinatorial library, consisting of 1,4-diazepan-2-ones, from the dye. After incubation of the library beads with fluorescently labeled target protein, confocal nanoscanning (CONA)^18^ is used to detect beads with bound protein. Once hit beads have been isolated, AIDA serves as a mass-tag to facilitate MS-decoding and as a reporter for a generic secondary binding assay for measuring the affinity of AIDA-tagged hit compounds with unlabeled target protein in homogenous solution.

Screening with fluorescently labeled LFA-1 I domain (LFA-1 ID) as target protein according to previous procedures for CONA on-bead screens yielded several inhibitors of various potencies with μm to nm dissociation constants (*K*_d_). However, close analysis of MS spectra during hit-bead decoding also revealed the presence of a precursor compound with exact mass of 427.20, corresponding to structure 1 (Figure 1 A). The local concentration or density of compounds on the surface of TentaGel beads is very high compared to standard screening concentrations in homogenous solutions. Consequently, the presence of binding-competent minor impurities can lead to target protein binding during on-bead screening. We therefore re-synthesized compound 1 to test its binding activity for fluorescently tagged LFA-1 ID using the same on-bead screening assay. Surprisingly, the resynthesized AIDA-alkyl diamine structure 1 showed very strong target protein binding, indicated by 100 % ring formation in the CONA screening image (Figure 1 B).

**Figure 1:**
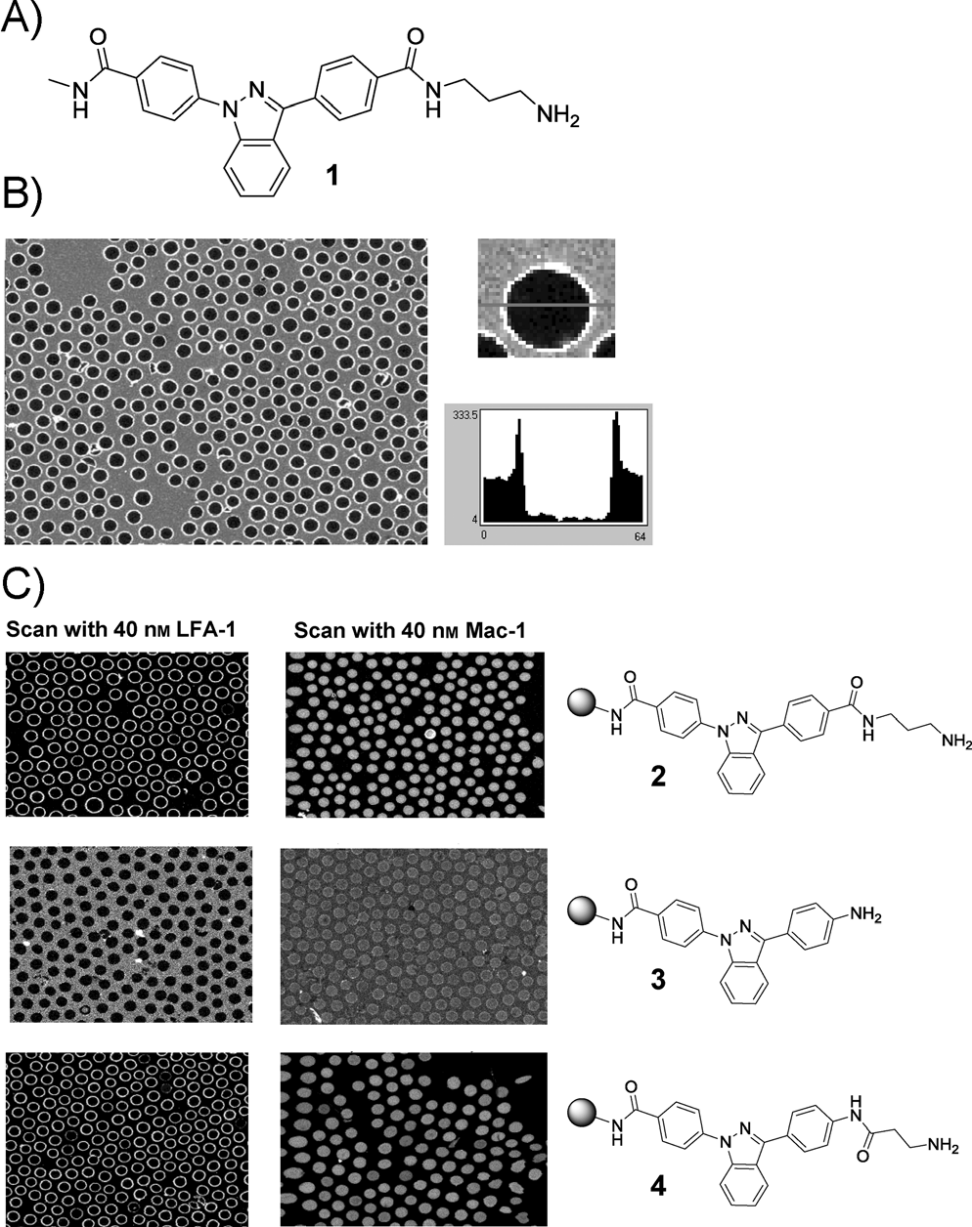
A) Structure of compound 1 (IBE-667) identified from screening of bead-based libraries with fluorescently tagged LFA-1 ID. B) CONA image of IBE-667 bearing beads incubated with 40 nm Cy5-LFA-1 ID. All beads in the overview show protein binding on the surface. One of the beads and the associated ring profile are shown on the right. C) On-bead structure–activity relationship and specificity testing: An amide-connected aminoalkyl group is required as substituent of the AIDA core for binding to LFA-1 ID. Fluorescently tagged Mac-1 ID does not bind to beads bearing compounds 1, 2, 3, or 4.

A further on-bead activity test showed that the interaction of compound **1** with LFA-1 ID was specific as no target protein binding was detected with the homologous MAC-1 ID (Figure 1 C). Furthermore, during SAR analysis, the presence of the aminoalkyl moiety proved to be an essential requirement for binding (Figure 1 C, compounds **2** versus compound **3**), whereas an inversion of the second amide bond and a shortening of the alkyl chain by one carbon atom still resulted in target-protein binding (Figure 1 C, compound **4**). To confirm the interaction found with compound **1** in homogenous solution, we performed fluorescence titrations, using unlabeled LFA-1 ID as receptor and AIDA-fluorescence emission intensity as the read-out. Upon incubation of AIDA compound **1** with LFA-1 ID, a concentration-dependent fluorescence intensity increase was detected. Curve fitting resulted in a dissociation constant of *K*_d_=52 nm (Figure 2 A). However, when soluble ICAM-1 was tested for its ability to compete with this interaction between LFA-1 ID and compound **1**, another satiable fluorescence intensity increase was observed. For compound **1** acting as an inhibitor, a decrease of the fluorescence intensity would be expected owing to replacement of compound **1** from the LFA-1 ID binding site by ICAM-1. Our data therefore suggested an agonistic behavior of compound **1** for the interaction of LFA-1 with ICAM-1. The fitted affinity of the LFA-1 ID/compound **1** complex to ICAM-1 was 76 nm (*K*_d_) for this interaction (Figure 2 B). For comparison, in the absence of any activation the low affinity conformation of LFA-1 binds ICAM-1 with an affinity of *K*_d_=1.5 mm.^25^

**Figure 2:**
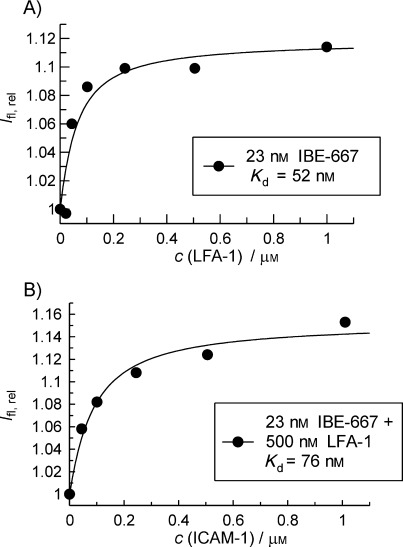
AIDA-based fluorescence titrations of A) IBE-667 with LFA-1 I domain and of B) IBE-667–LFA-1 ID complex with soluble ICAM-1. A) Addition of increasing amounts of LFA-1 I domain to 23 nm IBE-667 leads to an increase of AIDA fluorescence. Non-linear curve fitting of the experimental data produced a dissociation constant of *K*_d_=52±3 nm. B) Incubation of the preformed LFA-1 ID–IBE-667 complex with increasing amounts of soluble ICAM-1 resulted in a further AIDA fluorescence increase and revealed a dissociation constant for the [LFA-1 ID–IBE-667]–soluble ICAM-1 interaction of *K*_d_=76±2 nm.

With this in vitro characterization, we turned towards cellular assays to confirm that compound **1** was indeed an ICAM-1 binding enhancer for LFA-1. First, the ability of compound **1** to increase the binding of biotinylated soluble ICAM-1 to activated T-cells was investigated by FACS. After isolation of PBMCs and in vitro activation of T-cells, a dose dependent enhancement of biotinylated soluble ICAM-1 binding was detected in presence of compound **1** after staining with streptavidin-PE (Figure 3 A). This assay revealed a clear enhancement of the ICAM-1 binding affinity of LFA-1 in presence of compound **1**. Also, the compound **1** induced affinity enhancement of LFA-1 for ICAM-1 was completely reversed in presence of a known LFA-1 inhibitor binding in the Mevinolin binding pocket of the I-domain (Figure 3 B).^6^ Along with its ability to promote ICAM-1 binding to activated T-cells, compound **1** also enhanced aggregation of T-cells, a process which again is dependent on LFA-1/ICAM-1 interaction (Figure 3 C).^26^, ^27^ The dose-dependent induction of T-cell aggregation was completely blocked by a monoclonal antibody directed against LFA-1 (Figure 3 D). Based on these data we dubbed our newly identified LFA-1 agonist, *I*CAM-1 *b*inding *e*nhancer 667 (IBE-667).

**Figure 3:**
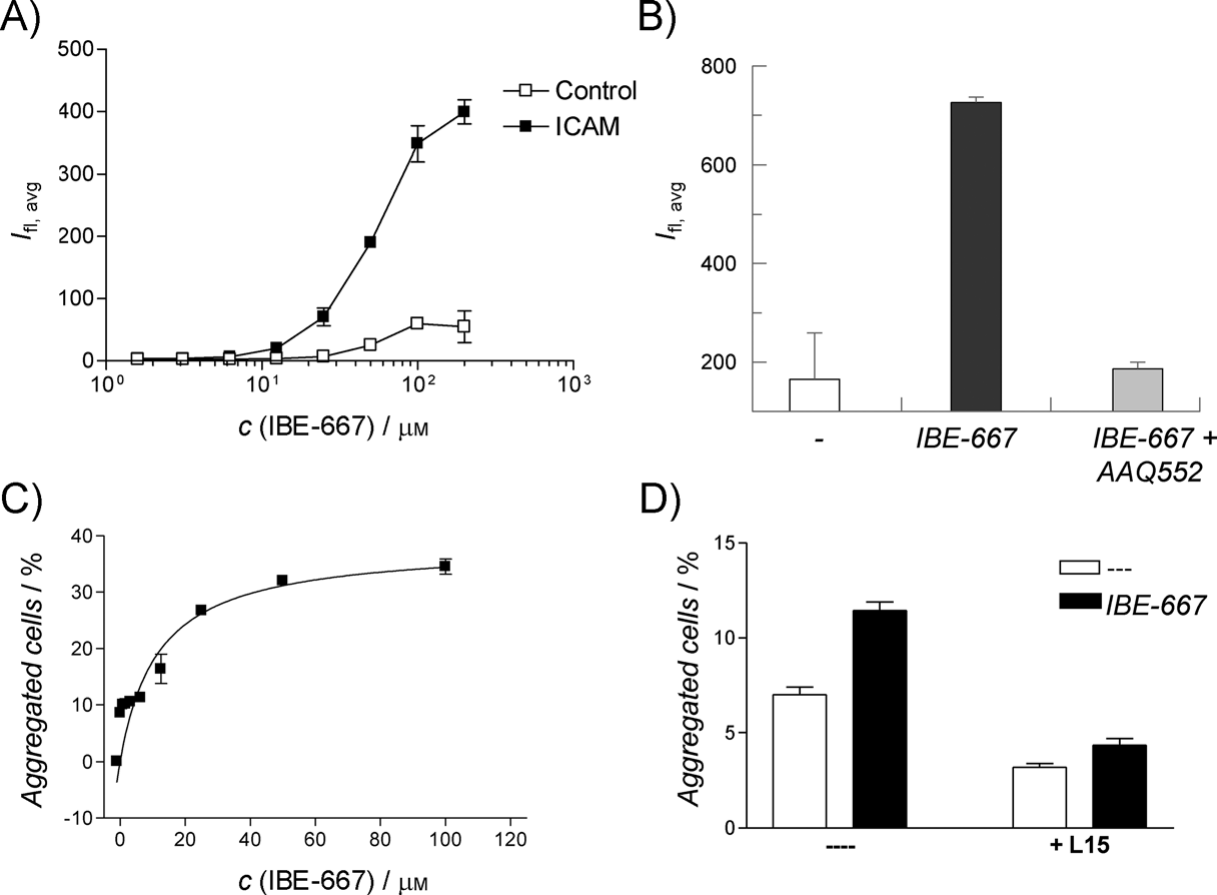
Activity of IBE-667 in cellular assays. A) IBE-667 increases the binding of biotinylated soluble ICAM-1 to activated T-cells as determined by staining with PE-streptavidin and FACS analysis. B) Soluble ICAM-1 binding to LFA-1 expressing activated T-cells cells in presence and absence of IBE-667 and LFA-1 inhibitor AAQ552. C) IBE-667 leads to a concentration dependent induction of T-cell aggregation after activation by anti CD3 and anti CD28 antibodies. D) IBE-667 induced T-cell aggregation can be completely reversed with an LFA-1 neutralizing antibody.

To further investigate the structural basis for the interaction of IBE-667 with LFA-1, we performed co-crystallization experiments. The complex LFA-1 I-domain/IBE-667 crystallized in an orthorhombic crystal form (*a*=45.3 Å, *b*=65.9 Å, *c*=133.6 Å, space group *I*222) with one complex per asymmetric unit. Diffraction data to a resolution of 1.8 Å were collected. The crystal structure (PDB-ID code 4IXD) revealed that IBE-667 does not bind to the MIDAS site of LFA-1 but instead binds to the mevinolin/lovastatin-binding pocket. The binding mode of IBE-667 consists of two hydrogen-bonding moieties (amide groups) that are flanking an extensive, rather planar aromatic moiety (the bis-phenylated indazole of AIDA) (Figure 4 A, B). Furthermore, the aminoalkyl group, which is responsible for the ICAM-1 binding enhancement, does not contribute interactions to the LFA-1 I-domain. The two flanking amide groups form direct hydrogen bonds to Tyr166-OH and Glu301-OE1, while the middle aromatic moiety interacts with the aromatic part of Tyr257 and the side chain of Lys287 (Figure 4 B, C). In particular, the aromatic interaction between the indazole and Tyr257 probably determines the binding mode in a crucial way. As the aminoalkyl group of IBE-667 does not make direct interactions with the LFA-1 I-domain, it is rather flexible, as judged by the weak electron density (Figure 4 C). A possible additional pseudo twofold related binding mode does not fit the electron-density equally well because of the asymmetry of the bond lengths between the indazole and the two neighboring phenyl moieties. Finally, an overlay of the complex structures of LFA-1 ID with a mevinolin derived inhibitor, LFA-878, and IBE-667 clearly demonstrates that the binding modes of these two compounds are different (Figure 4 D).

**Figure 4:**
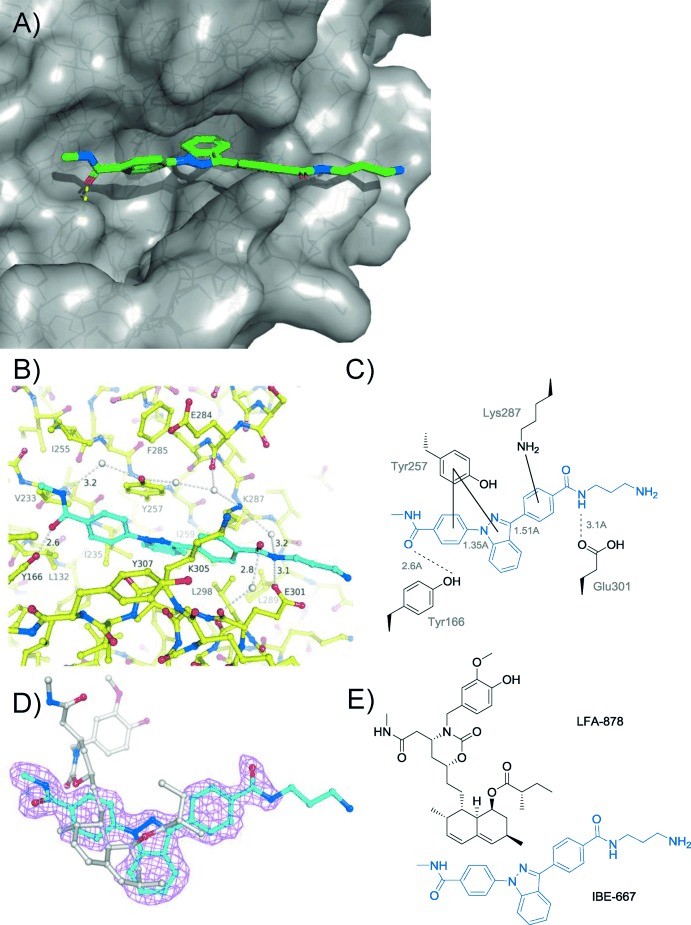
X-ray structure of IBE-667 bound to LFA-1 ID. A) Overview of co-crystal structure with IBE-667 in green, protein surface in gray, and the protein fold as solid ribbon. B) Interactions of IBE-667 in the mevinolin/lovastatin-binding pocket of LFA-1; LFA-1 carbon atoms are shown in yellow and the IBE-667 carbon atoms are shown in cyan. C) The main IBE-667 LFA-1 interactions. D) Final 2 *F*_o_−*F*_c_ electron density, contoured at 1σ, for IBE-667 (carbon atoms in cyan) and overlay with LFA-878 (carbon atoms in gray). E) Chemical structures of IBE-667 (blue) and the lovastatin-derived inhibitor LFA-878. Both compounds occupy the same binding pocket, but only IBE-667 acts as an agonist.

Overall, the binding mode of IBE-667 and the fact that the aminoalkyl chain does not interact directly with LFA-1 provides no obvious explanation for its ICAM-1 binding enhancement activity. A comparison of the LFA-1 ID IBE-667 structure to previously described low-, medium-, and high-affinity LFA-1 ID cystein mutants^9^ reveals that only small perturbations exist in the α7 helix of the LFA-1 ID. However, binding of mevinolin to LFA-ID, which occupies the same binding pocket as IBE-667, causes similarly subtle changes as found in the cystine mutant structures, but exhibits antagontistic behavior.^28^ Furthermore, similar to mevinolin, no changes are observed within the MIDAS binding site as a consequence of IBE-667 binding to LFA-1 ID. This suggests that the IBE-667 induced increased affinity of LFA-1 to ICAM-1 is attributable to dynamic kinetic effects, such as differences in on-rates. The additional charge introduced by the aminoalkyl group of IBE-667 could be a contributing factor.

In conclusion, we describe the identification of IBE-667, an ICAM-1 binding enhancing LFA-1 agonist by CONA on-bead screening of a tagged one-bead one-compound library. This compound, although a precursor of the original library design, once more shows the flexibility of on-bead screening for identification of biologically active ligands in a miniaturized and cost effective way. Furthermore, it also demonstrates the unique advantages of a tagged library approach, in the sense that the crucial confirmation of the interaction of the compound with the target protein in homogenous solution as well as an elucidation of the molecular mode of action is greatly facilitated by having a generic and sensitive fluorescence based assay. However, this result also demonstrates that the reporter group itself is not inert. Therefore, we have already designed and reported a next-generation tagged library approach, whereby the label is only introduced after the initial on-bead screening step, so that the label can only contribute to binding but not constitute the crucial interaction element.^21^ Finally, we believe that the insight gained from the LFA-1 ID IBE-667 co-crystal structure provides a valuable starting point for an optimized design of new LFA-1 agonists. LFA-1 agonists could have an intriguing potential for increasing the efficacy of tumor immunotherapy. The increase of LFA-1–ICAM-1 affinity by agonistic small molecules could compensate deficiencies in either LFA-1 expression or activation as seen in the LAD syndrome, a rare hereditary disease.

## Notes

miscellaneous_information
