# Supplementary material for: Identification and X-ray Co-crystal Structure of a Small-Molecule Activator of LFA-1-ICAM-1 Binding
Source: Angew Chem Int Ed Engl. 2014 Apr 1;53(17):4322–6. doi: 10.1002/anie.201310240 (PMC4314669; doi:10.1002/anie.201310240)
Supplement: Supplementary file 1 — miscellaneous_information [file anie0053-4322-sd1.pdf]

Angewandte Chemie
Eine Zeitschrift der Gesellschaft Deutscher Chemiker

Supporting Information
© Wiley-VCH 2014
69451 Weinheim, Germany

Identification and X-ray Co-crystal Structure of a Small-Molecule Activator of LFA-1-ICAM-1 Binding**

Martin Hintersteiner, Jörg Kallen, Mario Schmied, Christine Graf, Thomas Jung, Gemma Mudd, Steven Shave, Hubert Gstach, and Manfred Auer**

anie_201310240_sm_miscellaneous_information.pdf

Supporting online material

Contents
Part I Experimental Section
Part II: Comparison of IBE-667 co-crystal structure to previously published LFA-1 structures
Part III: Chemical Synthesis and Characterization

Part I Experimental Section

AIDA based fluorescence assay:

Steady state fluorescence measurements were performed on a SLM 8100C spectrofluorometer with excitation and emission wavelengths set to 336 and 400 nm, 1 nm slit width, respectively, using a 1 ml quartz cuvette, 10 mm optical path length, under constant stirring. IBE-667 at a concentration of 25 nM in a 50 mM TRIS/HCl buffer, pH 8.0 (with 100 mM KCl, 10 mM $MgCl_2$), containing 5% DMSO was titrated with LFA-1 ID to a final concentration of 500 nM, followed by a titration of the complex with ICAM-1(40 nM to 1 µM). After each addition of protein the solution was incubated for 15 min at 25°C with constant stirring. Dissociation constants were obtained by non-linear curve fitting in GraFit using a 1:1 binding model.

Binding of sICAM-1 to activated T cells:

Peripheral blood mononuclear cells (PBMC) were obtained from healthy donors and activated for 24 h to 72 h with anti-CD3 and anti-CD28 antibodies. Cells were incubated with the indicated compounds at various concentrations for 15 minutes on ice in PBS, 1 mM Ca^{2+}, 1 mM Mg^{2+} and 1% BSA. Biotinylated sICAM-1 was added at a final concentration of 2.5 µg/ml and incubated for 15 min on ice. Streptavidin-PE (*life* technologies) was added without further washing to a final concentration of 1 µg/ml for 15 min. Thereafter cells were washed twice in PBS and analyzed in duplicate by FACS. Results are expressed as percentage of sICAM-1 binding cells.

Aggregation of T cells

Activated T cells were split into two parts and labeled with either dihydroethidium or calcein AM (life-technologies) for 1 h at 37°C. Cells were washed and incubated together with or without the indicated compounds for 1 h at 37°C. Cells were analyzed by FACS and the portion of double-positive, aggregated cells was monitored. As a control, neutralizing anti-LFA-1 antibody L15 was used to block the interaction between LFA-1 and ICAM-1.

Co-crystallization and structure determination of the LFA-1 I domain/ IBE-667 complex

For the LFA-1 I domain, the construct comprising residues Lys127-Gly311 was used and purification was performed as reported previously.[28] The complex LFA-1 I-domain/**IBE-667** was prepared by adding the ligand (100mM solution in DMSO) to the protein solution (12.7 mg/ml, 10 mM $MgSO_4$, PBS pH 7.4). Briefly, 3 µl of LFA-1 I-domain/IBE-667 at 12.7 mg/ml (in 10 mM $MgSO_4$) were mixed with 2 µl of well solution (0.085 M sodium acetate, pH 4.6, 0.17 M ammonium acetate, 15% glycerol and 25.5% (w/v) PEG4000) and allowed to equilibrate against 0.7 ml of the latter at 20°C. The complex crystallized in an orthorhombic crystal form (a=45.3Å, b=65.9Å, c=133.6Å, in I222 with 1 complex/asymmetric unit). Diffraction data were collected to 1.8 Å at 20°C on a Mar345 imaging plate detector at the Swiss-Norwegian beamline of the European Synchrotron Facility (Grenoble, France). After data processing with the HKL software suite[29], initial phases were obtained by molecular replacement with X-PLOR Version 3.1 using 1XDG[30] as a search model. Manual rebuilding was done with O[31] and refinement with REFMAC5[32]. Five percent of the unique data were randomly selected for the calculation of Rfree. The final refinement model (one LFA-1 I domain including residues Gly128-Ile309, one **IBE-667** ligand, one magnesium ion, 299 water molecules) gave an R factor of 17.2% and Rfree = 19.9% (for all data in the resolution range 8-2.1 Å), with good stereochemistry (rms bond lengths = 0.007Å, rms bond angles = 0.97^o). The coordinates and structure factors have been deposited in the Protein Data Bank, www.rcsb.org (PDB ID code 4IXD).

***Part II:* Comparison of IBE-667 co-crystal structure to previously published LFA-1 structures**

Results

Structural comparison of the LFA-1 l-domain/IBE-667 structure to low, medium and high affinity I-domain cystine mutants produced by Shimaoka[9] et al. reveal small perturbations (largest RMSD of protein c-α positions compared to wild-type is 0.38Å) in the α7 helix of the LFA-1 l-domain, which increase affinity to ICAM-1. However, the opposite effect is also observable with the antagonist mevinolin, sharing the same binding pocket as IBE-667, causing a small, similar perturbation in the α7 helix (RMSDs of protein c-α positions compared to wildtype are 0.32 and 0.35 Å respectively). As noted by Kallen[28], no structural changes are observed within the MIDAS binding site upon mevinolin binding, the same holds true for IBE-667, suggesting the increased Kds of active mutants to ICAM-1 coordinated through this binding site are attributable to dynamic kinetic effects not captured in static form. This is partially backed up by the high activity mutants referred to above having improvements in Kd by changes in k_{on} and more pronounced in k_{off} rates.

Materials and methods:

Structural comparisons were performed using the PyMol molecular graphics system. The in-built MatchAlign algorithm brought highly active mutants (PDB IDs: 1MJN, 1MQA)[9] into spatial alignment with wildtype LFA1 I-domain (PDB ID: 3F74). Structural comparison of bound mevinolin/lovastatin to IBE-667 was achieved through similar structural alignments of lovastatin/KFA-1 I-domain (PDB ID: 1CQP) to the solved LFA-1 I-domain/IBE-667 structure.

With heightened activity levels of the I-domain cystine mutants seemingly explained by increasing disorder within the α7 helix, the newly presented LFA-1 l-domain/IBE-667 structure breaks with the trend, straightening and seemingly adding order to the α7 helix when compared to wildtype (see figure S2). Examination of the mevinolin-bound antagonist structure suggests an inactivation of LFA-1 with an increase on α7 helix order, the opposite observed with IBE-667 present (see figure S5).

Figure S1

α7 helix from alignments of LFA-1 l-domain/IBE-667 structure (yellow, with IBE-667 shown in cyan), medium affinity mutant with PDBID 1MJN (orange) and high affinity mutant with PDBID 1MQA (red), all aligned to wildtype LFA-1 I-domain structure with PDBID 3F74 (green).

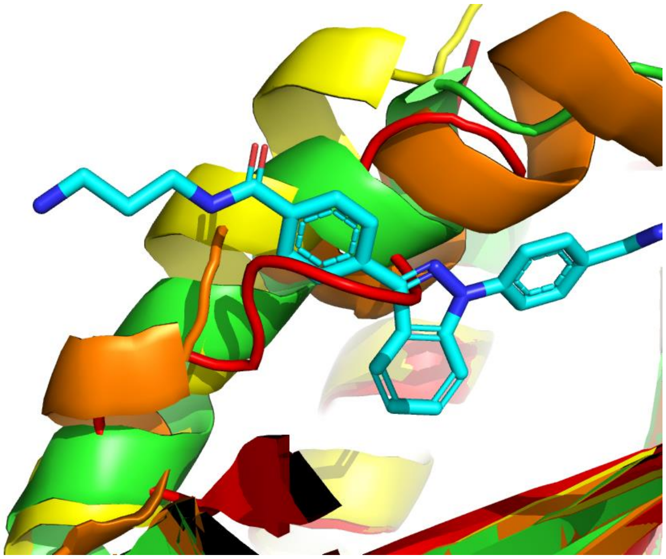

Figure S2

α7 helix from alignments of LFA-1 l-domain/IBE-667 structure (yellow, with IBE-667 shown in cyan), aligned to wildtype LFA-1 I-domain structure with PDBID 3F74 (green) – RMSD of all c-α positions between structures is 0.35 Å

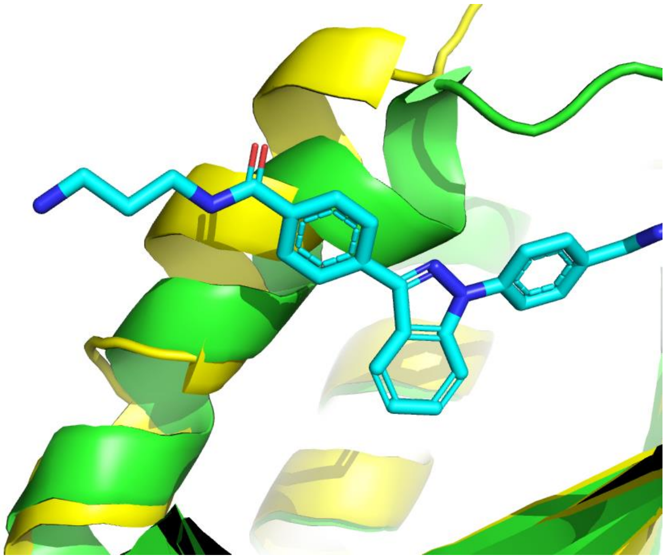

Figure S3

α7 helix from medium affinity mutant with PDBID 1MJN (orange) and high affinity mutant with PDBID 1MQA (red), both aligned to wild type LFA-1 I-domain structure with PDBID 3F74 (green) – RMSD of all c-α positions between structures is 0.28 Å and 0.38 Å, respectively.

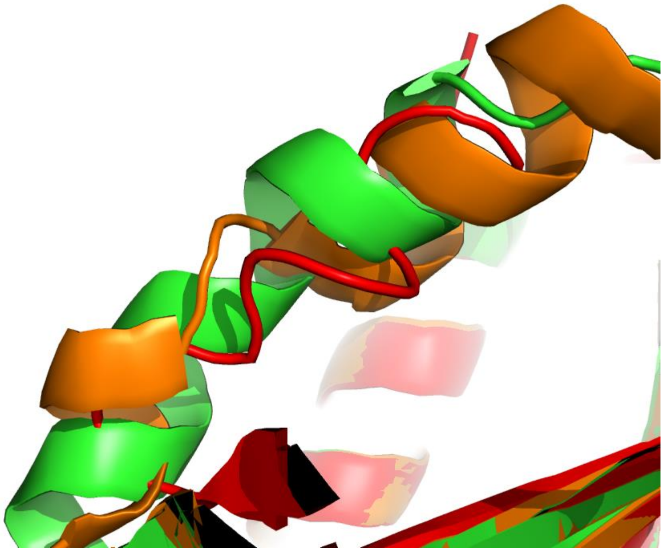

Figure S4

α7 helix from alignments of antagonist mevinolin-LFA-1 bound structure with PDBID 1CQP (magenta, with mevinolin also shown in magenta) to LFA-1 l-domain/IBE-667 structure (yellow, with IBE-667 also shown in yellow), RMSD of all c-α positions between structures is 0.38 Å.

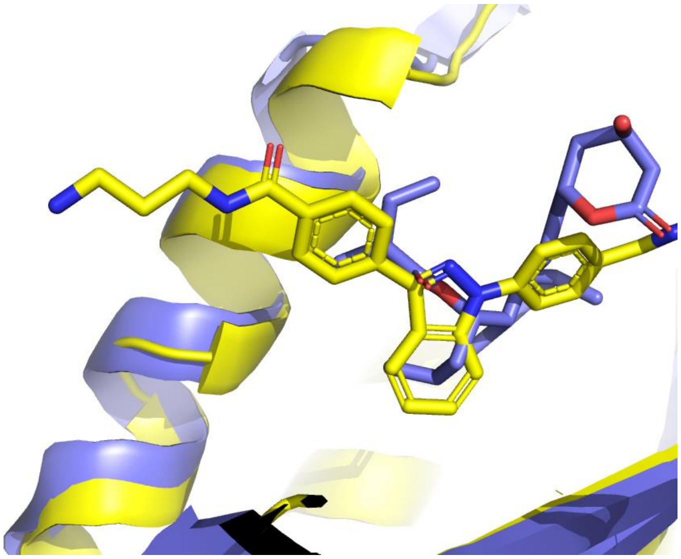

Figure S5

α7 helix from alignments of antagonist mevinolin-LFA-1 bound structure with PDBID 1CQP (magenta, with mevinolin also shown in magenta) to wildtype LFA-1 l-domain structure (green), RMSD of all c-α positions between structures is 0.32 Å.

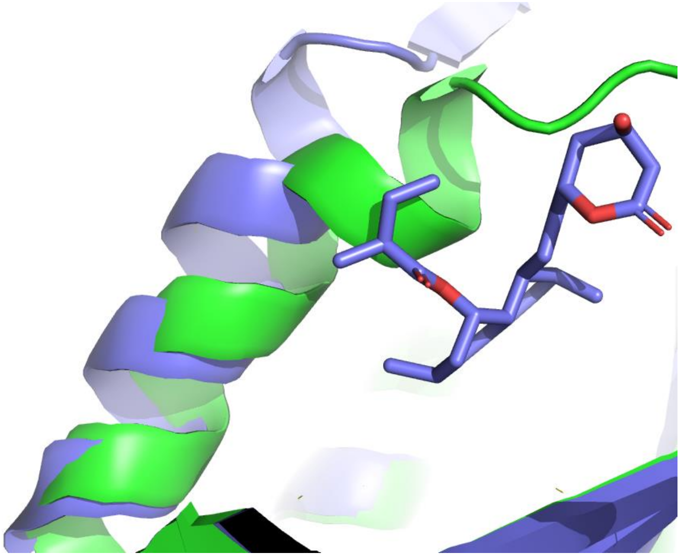

Figure S6

LFA-1 l-domain (yellow cartoon) with α7 helix (red cartoon), IBE-667 (blue spheres), MIDAS site Mg (green sphere) and ICAM-1 (cyan cartoon).

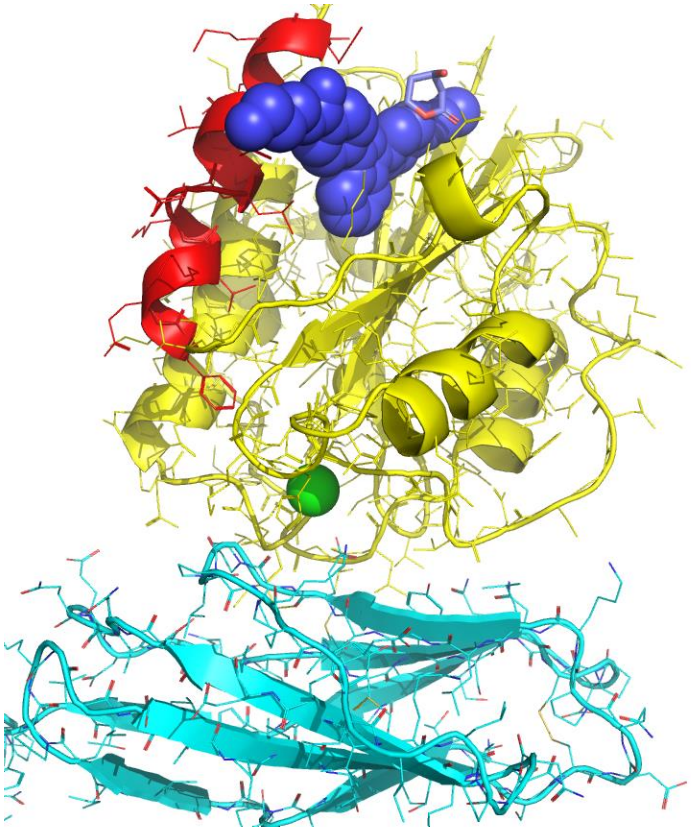

Part III: Chemical Synthesis and Characterization

HPLC-ESI-MS

Samples were analyzed on an HPLC-ESI-MS system consisting of a Finnigan Deka XP Plus ESI-MS detector connected to an Agilent 1100 capillary LC with a G1376A capillary pump, a G1377A Micro-autosampler and a G1325B DAD detector with micro flow cell. The system was run with a spray voltage of 5 kV, capillary temperature of 275°C, a capillary voltage of 15 V in single MS mode. The LC-system contained a Zorbax-SB-C18 0.5x35 mm 3.5 μm particle size column and was run with a gradient of solvent A: Water containing 0.1 % TFA and B: MeCN (LCMS grade) containing 0.1 % TFA. All analyses use standard gradient unless stated otherwise.

Standard Gradient: 2.5 min 0 % B, 22.5 min 0 – 100 % B, 25 min 100 – 0 % B, 28 min 0 % B at a flow rate of 200 μl/min.

HPLC-ESI-MS data were processed using the Xcalibur software package (version 2.0, Thermo Electron Corporation, MA, USA).

Analytical HPLC

HPLC analysis was performed on an Agilent 1100 series HPLC system, consisting of a quaternary pump (G1311A), a degasser (G1322A), an FLD detector (G1321A) and a DAD detector; column: Waters symmetry C8, 4.6 mm x 50 mm, 3.5 μm particle diameter size. Analyses were performed using a linear gradient of A: Water containing 0.1 % TFA and B: Acetonitrile (HPLC grade) containing 0.1 % TFA with a flow rate of 0.8 mL/min, Retention times (t_R) are denoted in minutes. All analyses use standard gradient unless stated otherwise.

Method A: 5 min 0 % B, 25 min 0 – 100 % B, 27.5 min 100 – 0 % B, 30 min 0 % B.

Method B: 0 min 20 % B, 5 min 20 % B, 30 min 20 – 40 % B, 33 min 40 – 100 % B, 35 min 100 – 20 % B.

Semi Preparative HPLC

Purification of mg quantities of peptides was carried out using a preparative HPLC system (Agilent 1100 prep-HPLC system), equipped with a preparative autosampler (G2260A), preparative scale pumps (G1361A), a fraction collector

(G1364B-prep) and a multiwavelength UV detector (G13658 MWD with preparative flow cell). Material was separated using either method A or B, which are outlined below.
Method A: Material was separated at a flow rate 20 mL/min on an Agilent RP-C-18 column (21.2 x 150 mm, 10 µm particle size), using a water/acetonitrile gradient and a detection wavelength of 210 nm. Solvent A: Water containing 0.1 % TFA; solvent B: Acetonitrile containing 0.1 % TFA. Gradient: 0 min 20 % B, 5 min 20 % B, 30 min 40 % B, 33 min 100 % B, 35 min 20 % B.
Method B: Material was separated at a flow rate 20 mL/min on a Waters X Bridge RP-C-18 column (18 x 100 mm, 5 µm particle size), using a water/acetonitrile gradient and a detection wavelength of 210 nm. Solvent A: Water, 0.1 % TFA; solvent B: Acetonitrile, 0.1 % TFA. Gradient: 5 min 0 % B, 45 min 80 % B, 50 min 80 % B, 55 min 0 % B, 60 min 0 % B.

Experimental Methods

The general procedure for synthesis of AIDA libraries has been described previously.[1,2] In short, the sequence consists of a) Loading standard 90 µm TentaGel® (NH_2) resin from Rapp Polymere (Tübingen, Germany) with 4-bromomethyl-3-nitrobenzoic acid as photocleavable linker. b) A methylamine substitution step is carried out to complete the photo-linker setup. c) The AIDA molecule including an Fmoc-protected diaminopropane spacer is immobilized on the beads as the first building block using (4-{3-{4-[N-3-[(9H-fluoren-9-yl)-methyloxycarbonylamino]-propyl]-aminocarbonyl}-phenyl-1H-indazol-1-yl} benzoic Acid as a building block. d) After Fmoc deprotection library synthesis is started using the first variable building block.

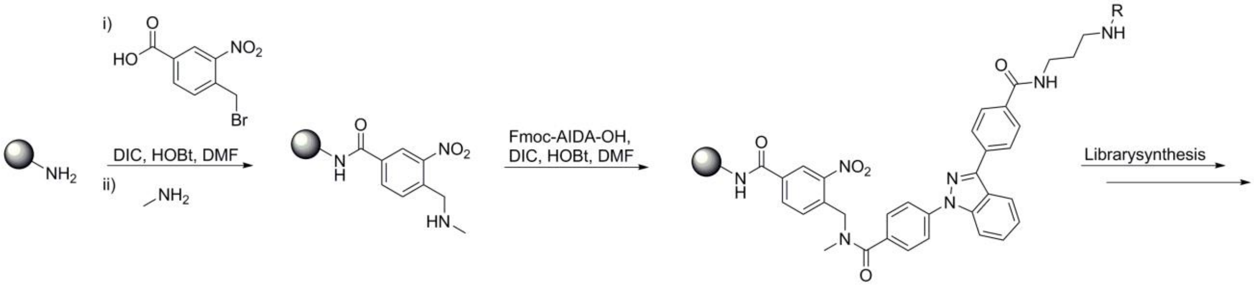

6 R = Fmoc "TG-PL-AIDA-Fmoc"
7 R = H "TG-PL-AIHA-OH"

4-{3-[4-(methoxycarbonyl)-phenyl]-1H-indazol-1yl}-benzoic Acid (CAS 279249-34-2):
The AIDA precursor was synthesized according to literature described procedures:
1. Gladstone, W. A. F. and Norman, R. O. C. J. Chem. Soc. (1965), 3048-52;
2. Gladstone, W. A. F. and Norman, R. O. C. J. Chem. Soc. (1965), 5177-82;
3. Gladstone, W. A. F. et al. J. Chem. Soc. (1966), 1781-4;
4. Iffland, D. C. et al. J. Am. Chem. Soc. (1961) 83, 747

4-[3-[4-(3-Aminopropylcarbamoyl)phenyl]indazol-1-yl]benzoic acid (CAS 279249-36-4):

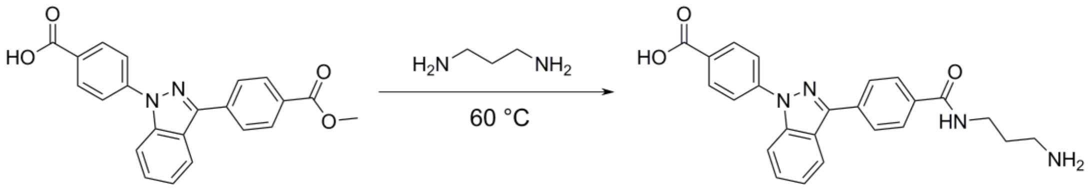

4-[3-(4-Methoxycarbonylphenyl)indazol-1-yl]benzoic acid (1.00 g; 2.69 mmol; CAS 279249-34-2) was added to 1,3-diaminopropane (10 mL) and the mixture was heated to 60 °C for 6 h. The reaction was followed by TLC and once disappearance of the starting material was observed, excess diamine was removed under reduced pressure to give a viscous orange oil. To this was added methanol (15 mL) and the mixture heated to reflux for 10 min. During this time a white solid precipitated, which was collected by filtration and washed with diethyl ether. The residual

material was dried under vacuum to give the title compound as a white solid (800 mg, 70%). HPLC-MS (ESI) t_R: 11.34; Exact mass calcd for $C_{24}H_{23}N_4O_3$ $[M+H]^+$: 415.18, found: 415.11.

4-[3-[4-(2-Aminoethylcarbamoyl)phenyl]indazol-1-yl]benzoic acid:

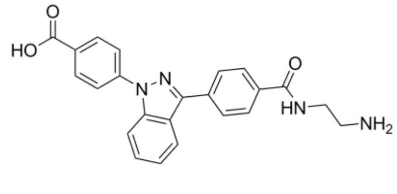

The title compound was prepared from 4-[3-(4-methoxycarbonyl - phenyl)indazol-1-yl]benzoic acid (2.00 g; 5.34 mmol; CAS 279249-34-2) using the same procedure as for the propyl derivative outlined above but exchanging 1,3-diaminopropane for 1,2-diaminoethane (20 mL) to give the amino acid as a white solid (1.94 g, 88%). HPLC-MS (ESI) t_R: 11.04; Exact mass calcd for $C_{23}H_{21}N_4O_3$ $[M+H]^+$: 401.16, found: 401.11.

4-[3-[4-[3-(9H-Fluoren-9-ylmethoxycarbonylamino)propylcarbamoyl]phenyl]indazol-1-yl]benzoic acid (CAS 279249-37-5):

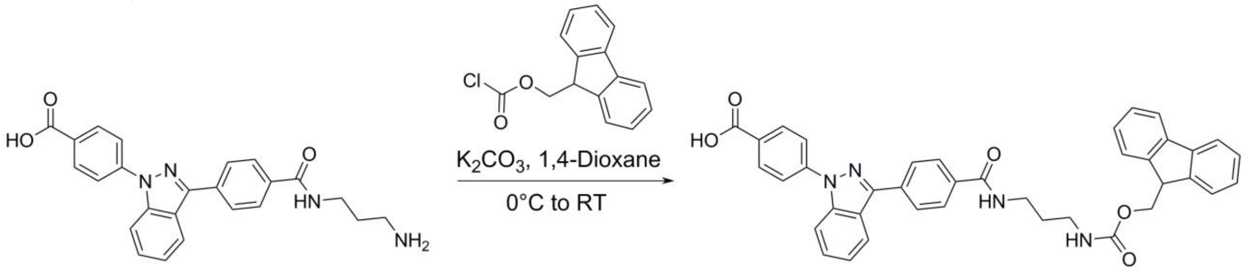

4-[3-[4-(3-Aminopropylcarbamoyl)phenyl]indazol-1-yl]benzoic acid (400 mg; 0.93 mmol; CAS 279249-36-2) was stirred in 1,4-dioxane/water (1:1, 30 mL). To this was added potassium carbonate (257 mg; 1.86 mmol) and the mixture cooled to 0 °C. Fmoc chloride (265 mg; 1.02 mmol) in 1,4-dioxane (5 mL) was added slowly then the mixture was allowed to warm to room temperature and was stirred overnight. The mixture was washed with diethyl ether then acidified to pH1 with 2M HCl which caused a precipitate to form. This was filtered off and washed with diethyl ether then dried to give the title compound as a white solid (493 mg, 83%). HPLC-MS (ESI) t_R: 16.36; Exact mass calcd for $C_{39}H_{33}N_4O_5$ $[M+H]^+$: 637.24, found: 637.14.

4-[3-[4-[2-(9H-Fluoren-9-yl)methoxycarbonylamino)ethylcarbamoyl]phenyl]indazol-1-yl]benzoic acid:

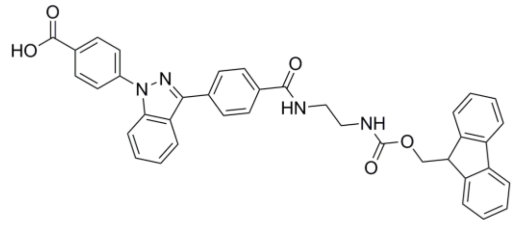

The title compound was prepared from 4-[3-[4-(3-amino-ethylcarbamoyl) phenyl]indazol-1-yl]benzoic acid (1.00 g; 2.50 mmol) using the same procedure as for the propyl derivative outlined above to give the Fmoc protected amine as a white solid (1.05 g; 1.69 mmol, 68%). HPLC-MS (ESI) t_R: 16.03; Exact mass calcd for $C_{38}H_{31}N_4O_5$ $[M+H]^+$: 623.23, found: 623.09.

9H-Fluoren-9-ylmethyl N-[3-[[4-[1-[4-(methylcarbamoyl)phenyl]indazol-3-yl]benzoyl]amino]propyl]- carbamate :

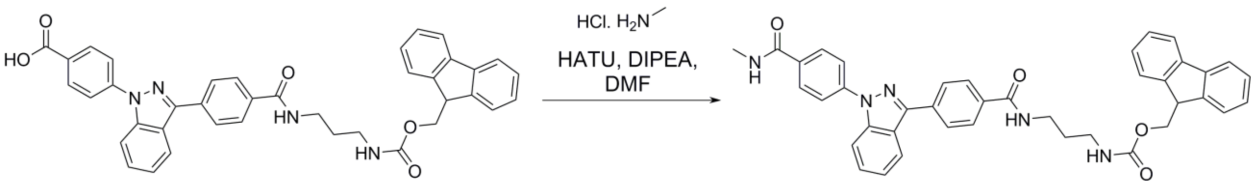

4-[3-[4-[3-(9H-Fluoren-9-ylmethoxycarbonylamino)propylcarbamoyl]phenyl]indazol-1-yl]benzoic acid (100 mg; 0.16 mmol; CAS 279249-37-5) and methylamine hydrochloride (13 mg; 0.19 mmol) were stirred in DMF under argon and the mixture cooled to 0°C. HATU (72.2 mg; 0.19 mmol) and DIPEA (219 µL; 1.26 mmol) were added and the mixture was allowed to warm to rt and stirred overnight. The product was precipitated by adding excess of water. The white precipitate was washed with ethyl acetate and dried under vacuum (27 mg; 26%). HPLC-MS (ESI) t_R: 15.83; Exact mass calcd for $C_{40}H_{36}N_5O_4$ $[M+H]^+$: 650.28, found: 650.14.

9H-Fluoren-9-ylmethyl N-[3-[[4-[1-[4-(methylcarbamoyl)phenyl]indazol-3-yl]benzoyl]amino]ethyl] carbamate:

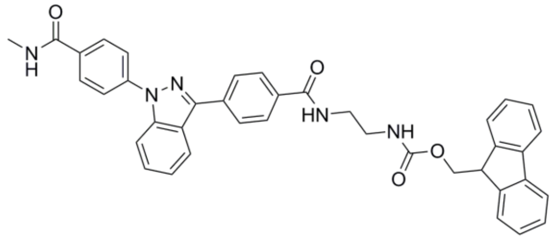

The title compound was prepared from 4-[3-[4-[2-(9H-fluoren-9-ylmethoxycarbonylamino)ethylcarbamoyl]phenyl] -indazol-1-yl]benzoic acid (400 mg; 0.64 mmol) using the same procedure as for the propyl derivative outlined above to give the methyl amide as a white solid (393 mg, 97%). HPLC-MS (ESI) t_R: 15.46; Exact mass calcd for $C_{39}H_{34}N_5O_4$ $[M+H]^+$: 636.26, found: 636.12.

N-(3-Aminopropyl)-4-[1-[4-(methylcarbamoyl)phenyl]indazol-3-yl]benzamide :

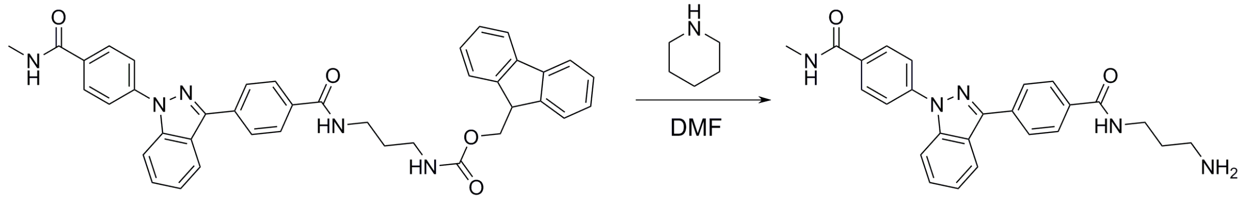

9H-Fluoren-9-ylmethyl N-[3-[[4-[1-[4-(methylcarbamoyl)phenyl]indazol-3-yl]benzoyl]amino]propyl]carba-mate (27.00 mg; 0.0416 mmol) was stirred in a solution of 20% piperidine in DMF (2 mL) for 3 h. Solvents were removed under reduced pressure and water used to precipitate the product. A white solid was filtered off, washed with diethyl ether and dried. The residual material was purified using semi- preparative RP HPLC (Method A) as described above to yield the title compund as a white solid (3.95 mg, 23%); HPLC-MS (ESI) t_R: 10.95; Exact mass calcd for $C_{25}H_{26}N_5O_2$ $[M+H]^+$: 428.21, found: 428.14; RP HPLC t_R: 14.79 (99%, 220 nm), Method A; ^{1}H NMR (500 MHz, DMF-d_7) δ 8.63 (1H, d, *J* = 4.5 Hz), 8.44 (2H, br s), 8.33 (2H, d, *J* = 8.3 Hz), 8.31 - 8.27 (2H, m), 8.24 (2H, d, *J* = 8.7 Hz), 8.22 – 8.19 (2H, m), 8.10 (2H, d, *J* = 8.6 Hz), 8.08 (2H, d, *J* = 8.7 Hz), 7.66 (1H, ddd, *J* = 8.4, 7.1, 0.9 Hz), 7.50 – 7.44 (1H, m), 3.60 (2H, q, *J* = 6.3 Hz), 3.24 (2H, t, *J* = 7.3 Hz), 2.95 (3H, d, J = 4.5 Hz), 2.12 (2H, quin, *J* = 6.9 Hz); ^{13}C NMR (125.8 MHz, DMF-d_7) δ 166.8 (C), 166.2 (C), 145.2 (C), 142.0 (C), 140.3 (C), 135.7 (C), 134.4 (C), 133.0 (C), 128.8 (CH), 128.1 (CH), 127.4 (CH), 123.2 (C), 123.1 (CH), 122.0 (CH), 121.7 (CH), 111.5 (CH), 37.7 (CH_2), 36.6 (CH_2), 27.9 (CH_2), 26.1 (CH_3).

N-(2-Aminoethyl)-4-[1-[4-(methylcarbamoyl)phenyl]indazol-3-yl]benzamide:

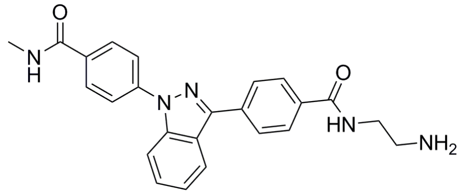

The title compound was prepared from 9H-fluoren-9-ylmethyl N-[3-[[4-[1-[4-(methylcarbamoyl)phenyl]indazol-3-yl]benzoyl] -amino]ethyl] carbamate (50.00 mg; 0.0787 mmol) following the same procedure as for the propyl derivative outlined above and purified using semi preparative RP HPLC (Method A) to give the amine as a white solid (11.05 mg, 34%); HPLC-MS (ESI) t_R: 10.95; Exact mass calcd for $C_{24}H_{24}N_5O_2$ $[M+H]^+$: 414.19, found: 414.09; RP HPLC t_R: 13.71 (95%, 220 nm), Method B; ^{1}H NMR (500 MHz, DMF-d_7) δ 9.09 (1H, t, *J* = 5.4 Hz), 8.63 (2H, d, J = 4.5 Hz), 8.58 (2H, br s), 8.33 (2H, d, *J* = 8.3 Hz), 8.31 – 8.27 (2H, m), 8.26 – 8.20 (4H, m), 8.13 – 8.06 (4H, m), 7.66 (t, *J* = 7.4 Hz), 7.50 – 7.45 (1H, m), 3.83 (2H, q, *J* = 5.6 Hz), 3.41 (2H, t, *J* = 5.6 Hz), 2.95 (3H, d, J = 4.6 Hz); ^{13}C NMR (125.8 MHz, DMF-d_7) δ 167.1 (C), 166.3 (C), 145.3 (C), 142.2 (C), 140.5 (C), 135.9 (C), 134.4 (C), 133.2 (C), 129.0 (CH), 128.4 (CH), 128.3 (CH), 127.5 (CH), 123.4 (C), 123.3 (CH), 122.2 (CH), 121.8 (CH), 111.6 (CH), 40.0 (CH_2), 38.0 (CH_2), 26.3 (CH_3).

4-[3-[4-(3-aminopropanoylamino)phenyl]indazol-1-yl]benzamide

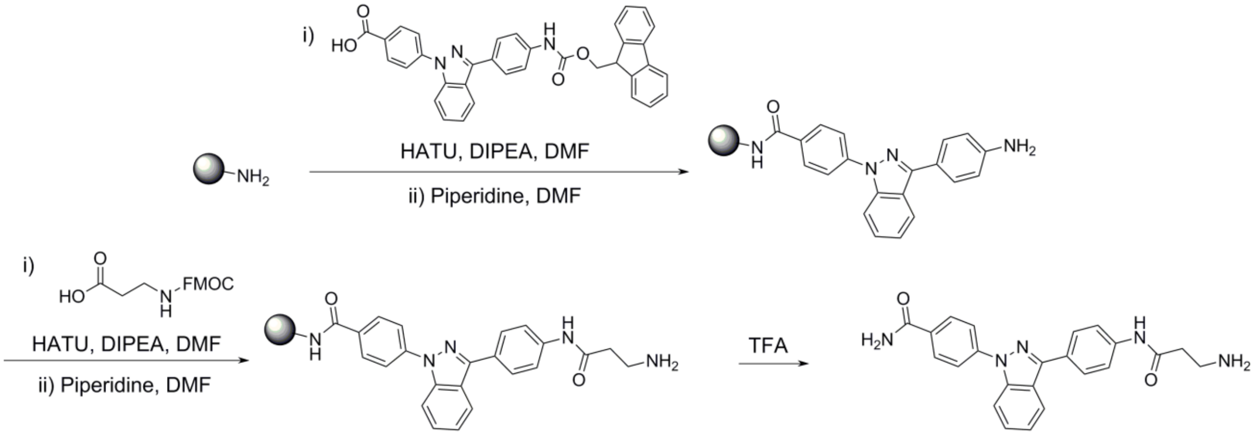

The title compound was synthesized using Fmoc solid phase peptide synthesis. Tentagel® S RAM resin (Rapp Polymere) was preswollen in DMF then to this was added a solution of 5 equiv. amino acid, 5 equiv. HATU and 10 equiv. DIPEA in DMF. The reaction mixture was shaken for 1 h followed by washing repeatedly with DMF then DCM. Fmoc group removal was carried out by shaking the resin in a solution of 20% piperidine in DMF, followed by excessive washing with DMF and DCM. Cleavage of the final compound from the solid support was performed with TFA for 2 h. The solution was then concentrated to dryness and the residue purified using RP HPLC (Method B) to yield the title compound as a white solid. HPLC-MS (ESI) t_R: 10.79; Exact mass calcd for $C_{23}H_{22}N_5O_2$ $[M+H]^+$: 400.18, found: 400.11; RP HPLC t_R: 11.71 (98%, 220 nm), Method A; ^{1}H NMR (500 MHz, DMF-d_7) δ 10.60 (1H, s), 8.43 (2H, br s), 8.30 - 8.26 (3H, m), 8.16 – 8.13 (2H, m), 8.10 (1H, d, *J* = 8.6 Hz), 8.08 – 8.05 (2H, m), 7.94 (2H, d, *J* = 8.7 Hz), 7.64 (1H, ddd, *J* = 8.4, 7.1, 1.0 Hz), 7.45 – 7.43 (1H, m), 3.49 (2H, t, *J* = 6.5 Hz), 3.04 (2H, t, *J* = 6.5 Hz); ^{13}C NMR (125.8 MHz, DMF-d_7) δ 169.1 (C), 167.5 (C), 145.9 (C), 142.4 (C), 140.2 (C), 139.9 (C), 132.5 (C), 129.3 (CH), 128.1 (CH), 128.0 (C), 127.9 (C), 123.2 (C), 122.8 (CH), 121.8 (CH), 121.6 (CH), 119.6 (CH), 111.3 (CH), 36.1 (CH_2), 33.7 (CH_2).

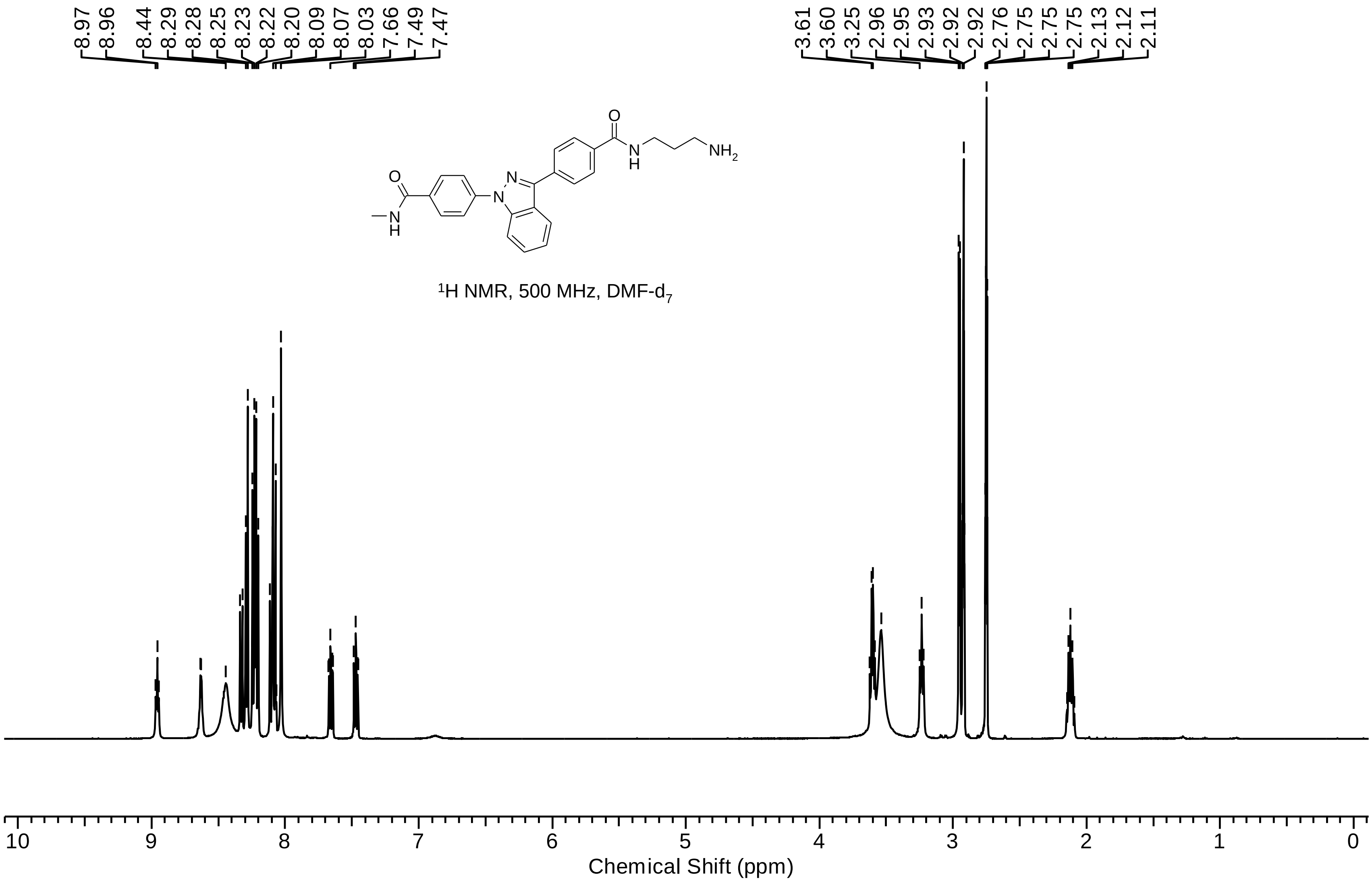

8.97
8.96
8.44
8.29
8.28
8.25
8.23
8.22
8.20
8.09
8.07
8.03
7.66
7.49
7.47
3.61
3.60
3.25
2.96
2.95
2.93
2.92
2.92
2.76
2.75
2.75
2.75
2.13
2.12
2.11
O
N
N
N
H
NH2
O
N
H
1H NMR, 500 MHz, DMF-d7
10
9
8
7
6
5
4
3
2
1
0
Chemical Shift (ppm)

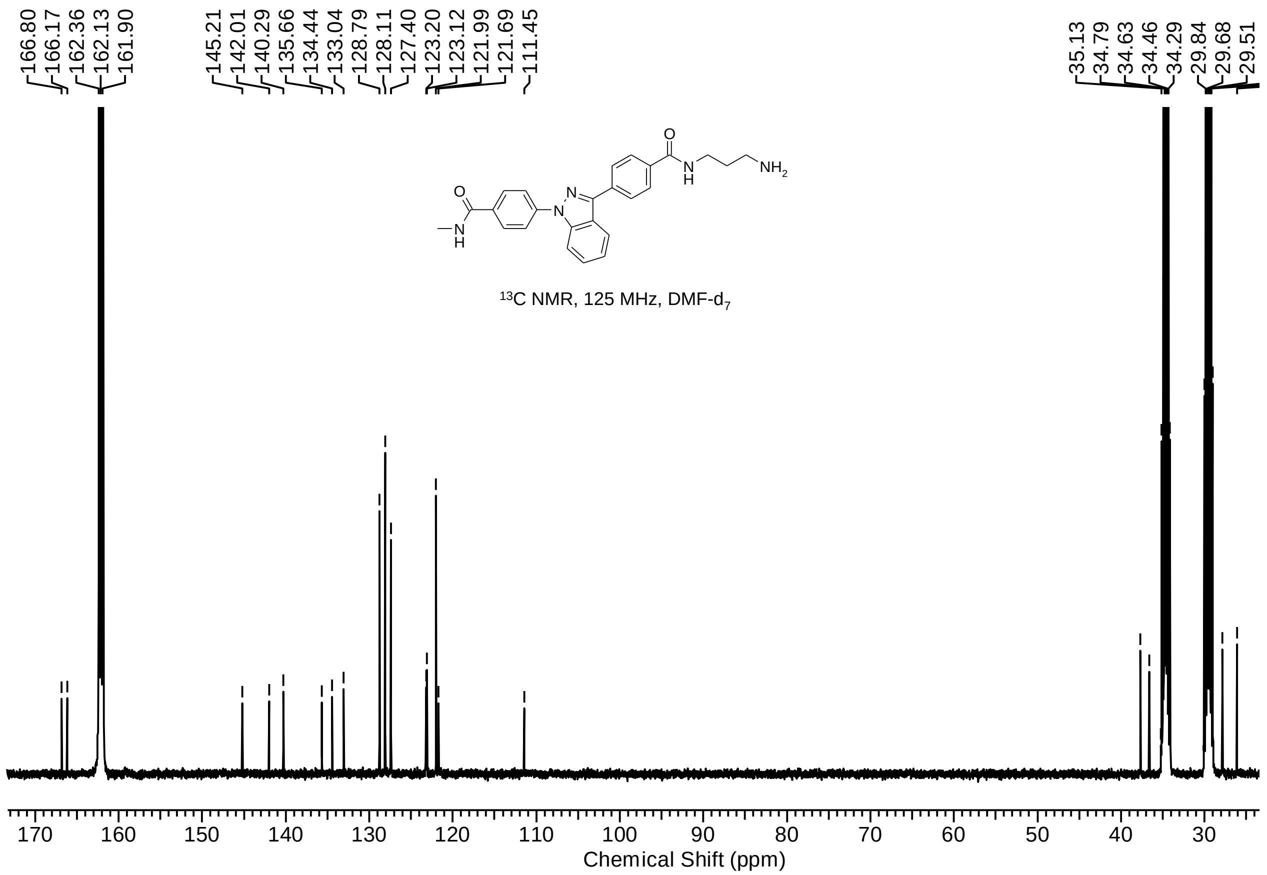

166.80
166.17
162.36
162.13
161.90
145.21
142.01
140.29
135.66
134.44
133.04
128.79
128.11
127.40
123.20
123.12
121.99
121.69
111.45
35.13
34.79
34.63
34.46
34.29
29.84
29.68
29.51
O
N
H
N
N
O
N
H
NH2
13C NMR, 125 MHz, DMF-d7
170
160
150
140
130
120
110
100
90
80
70
60
50
40
30
Chemical Shift (ppm)

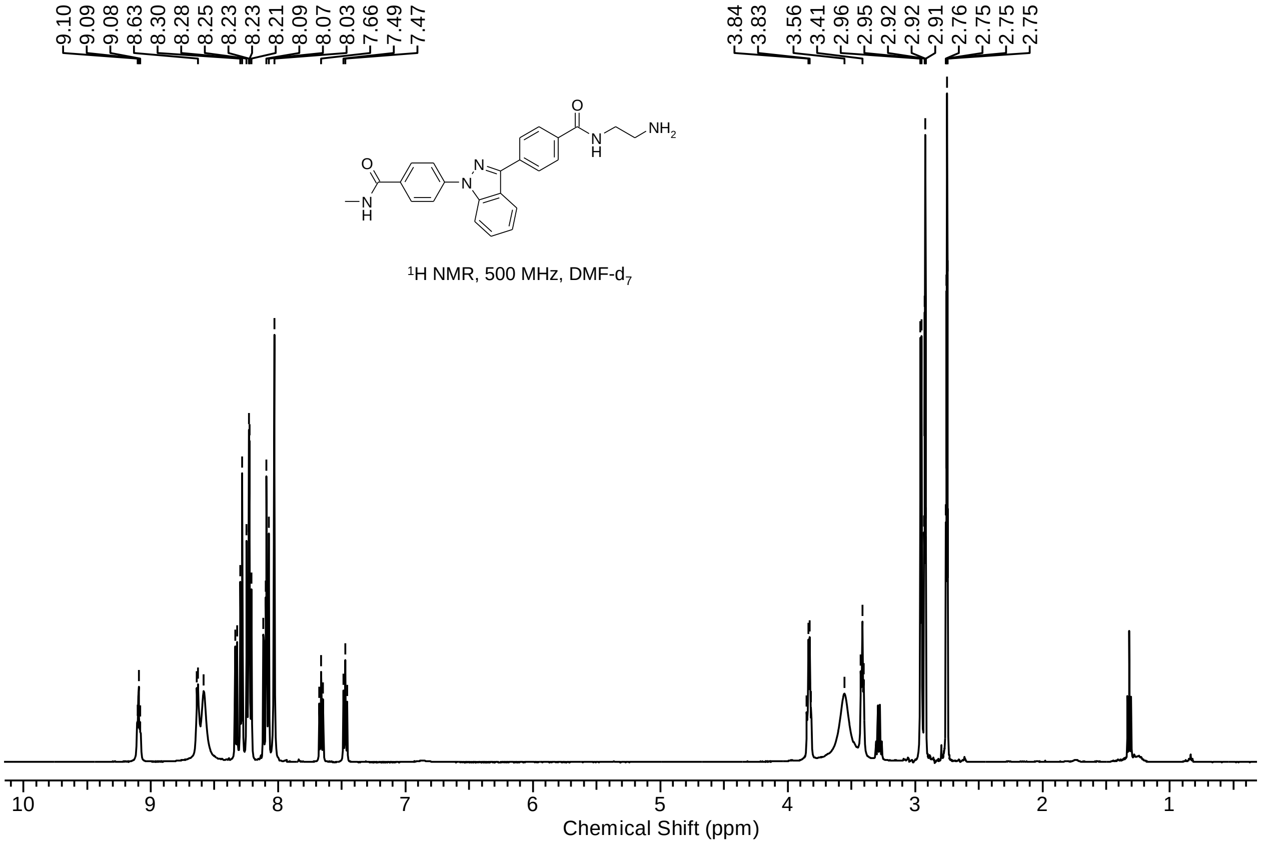
9.10
9.09
9.08
8.63
8.30
8.28
8.25
8.23
8.23
8.21
8.09
8.07
8.03
7.66
7.49
7.47
3.84
3.83
3.56
3.41
2.96
2.95
2.92
2.92
2.91
2.76
2.75
2.75
2.75
O
N
H
N
N
O
N
H
NH2
1H NMR, 500 MHz, DMF-d7
10
9
8
7
6
5
4
3
2
1
Chemical Shift (ppm)

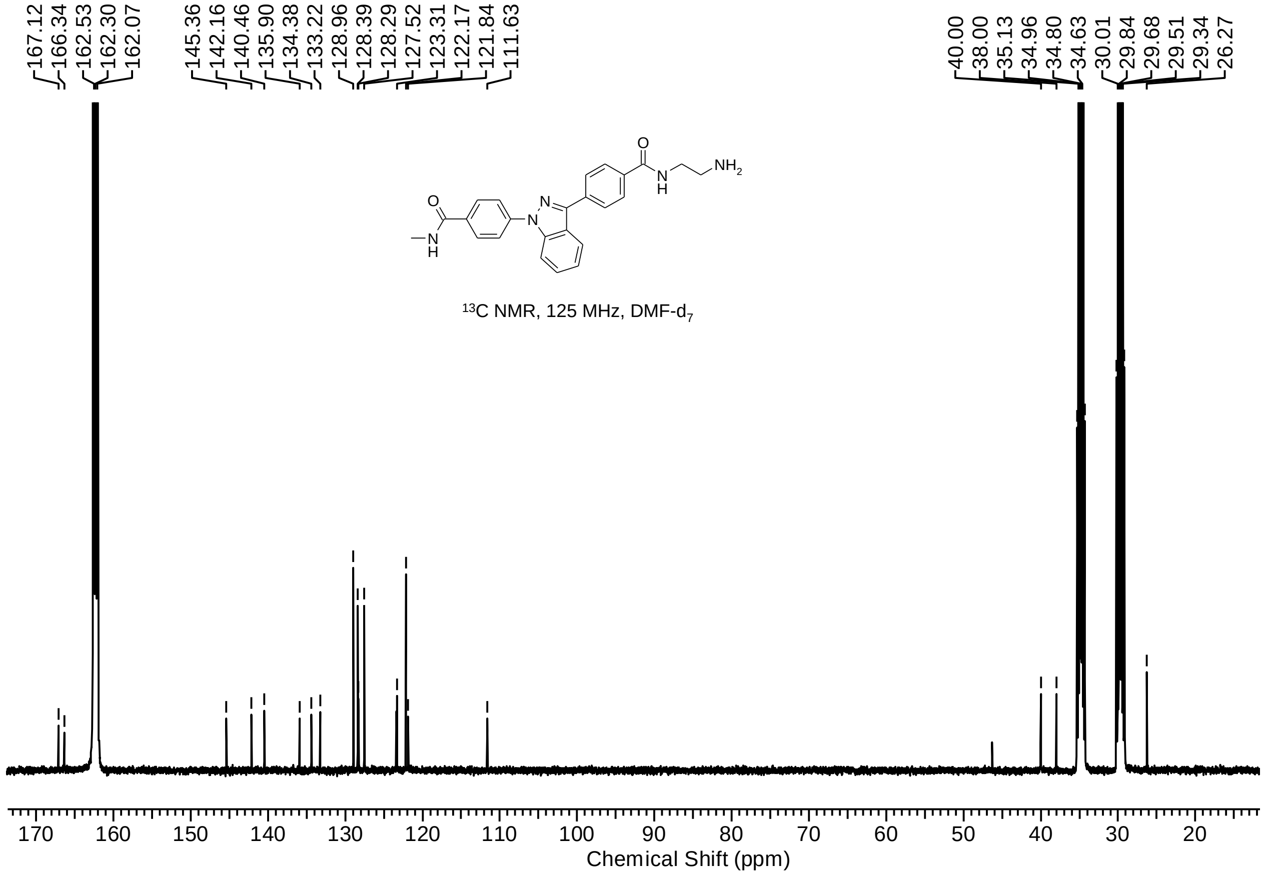

167.12
166.34
162.53
162.30
162.07
145.36
142.16
140.46
135.90
134.38
133.22
128.96
128.39
128.29
127.52
123.31
122.17
121.84
111.63
40.00
38.00
35.13
34.96
34.80
34.63
30.01
29.84
29.68
29.51
29.34
26.27
O
N
H
N
N
O
N
H
NH2
13C NMR, 125 MHz, DMF-d7
170
160
150
140
130
120
110
100
90
80
70
60
50
40
30
20
Chemical Shift (ppm)

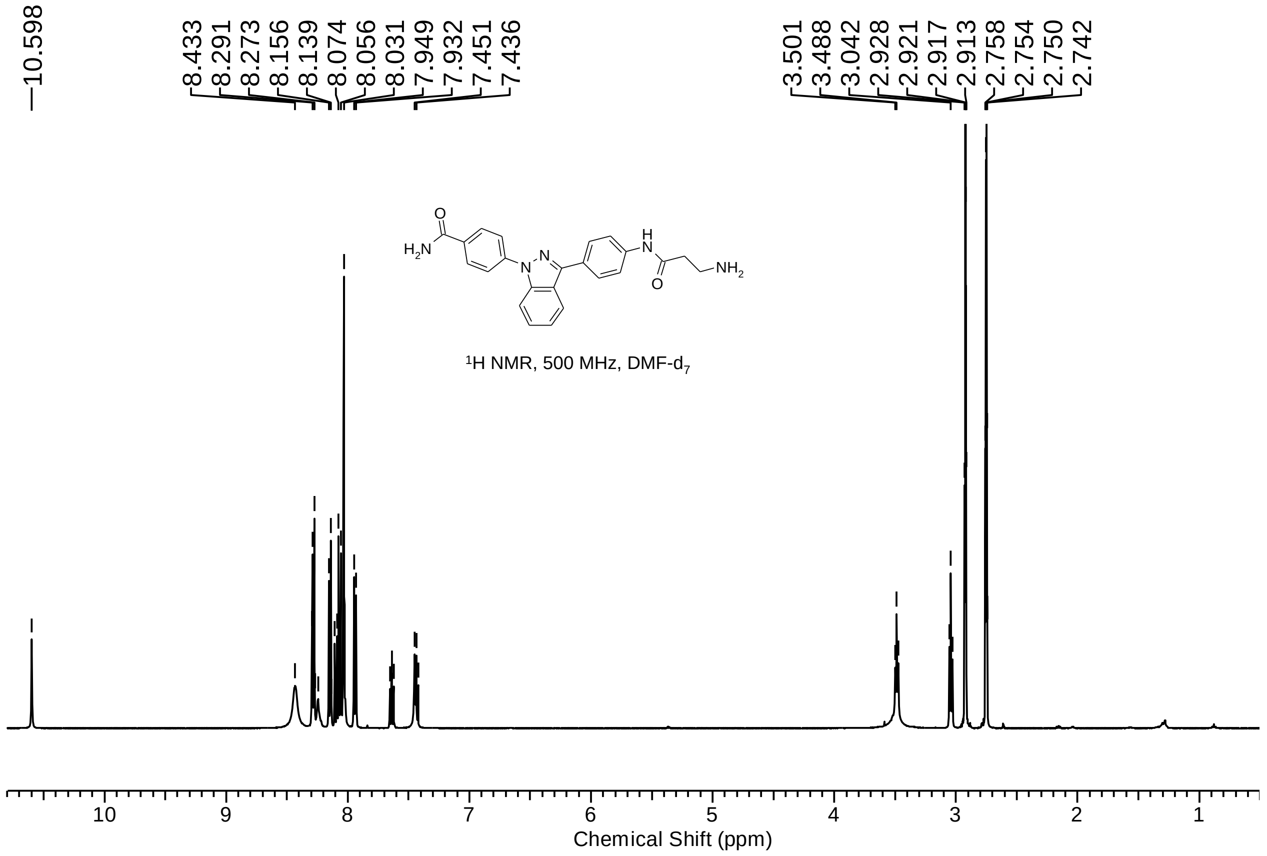
10.598
8.433
8.291
8.273
8.156
8.139
8.074
8.056
8.031
7.949
7.932
7.451
7.436
3.501
3.488
3.042
2.928
2.921
2.917
2.913
2.758
2.754
2.750
2.742
1H NMR, 500 MHz, DMF-d7
10
9
8
7
6
5
4
3
2
1
Chemical Shift (ppm)

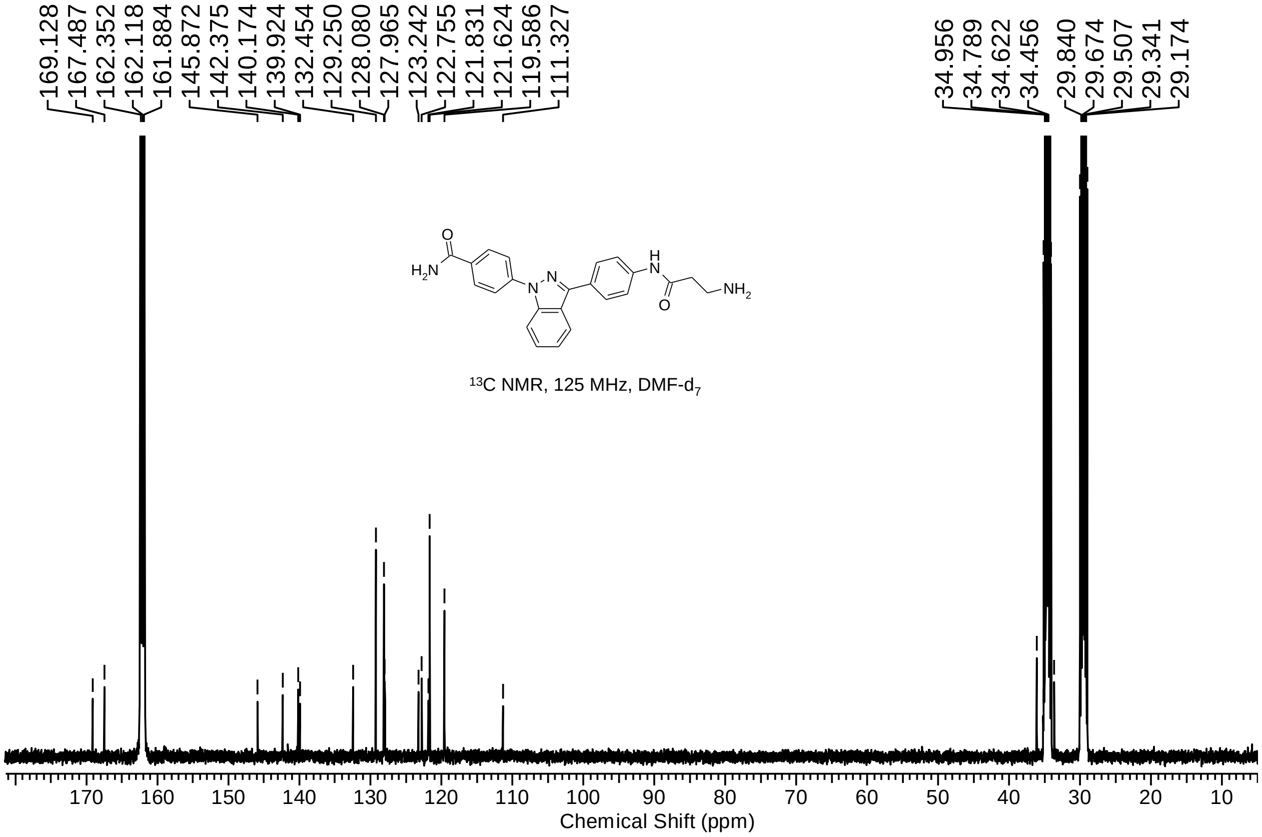
169.128
167.487
162.352
162.118
161.884
145.872
142.375
140.174
139.924
132.454
129.250
128.080
127.965
123.242
122.755
121.831
121.624
119.586
111.327
34.956
34.789
34.622
34.456
29.840
29.674
29.507
29.341
29.174
O
H2N
N
N
H
N
O
NH2
13C NMR, 125 MHz, DMF-d7
170
160
150
140
130
120
110
100
90
80
70
60
50
40
30
20
10
Chemical Shift (ppm)

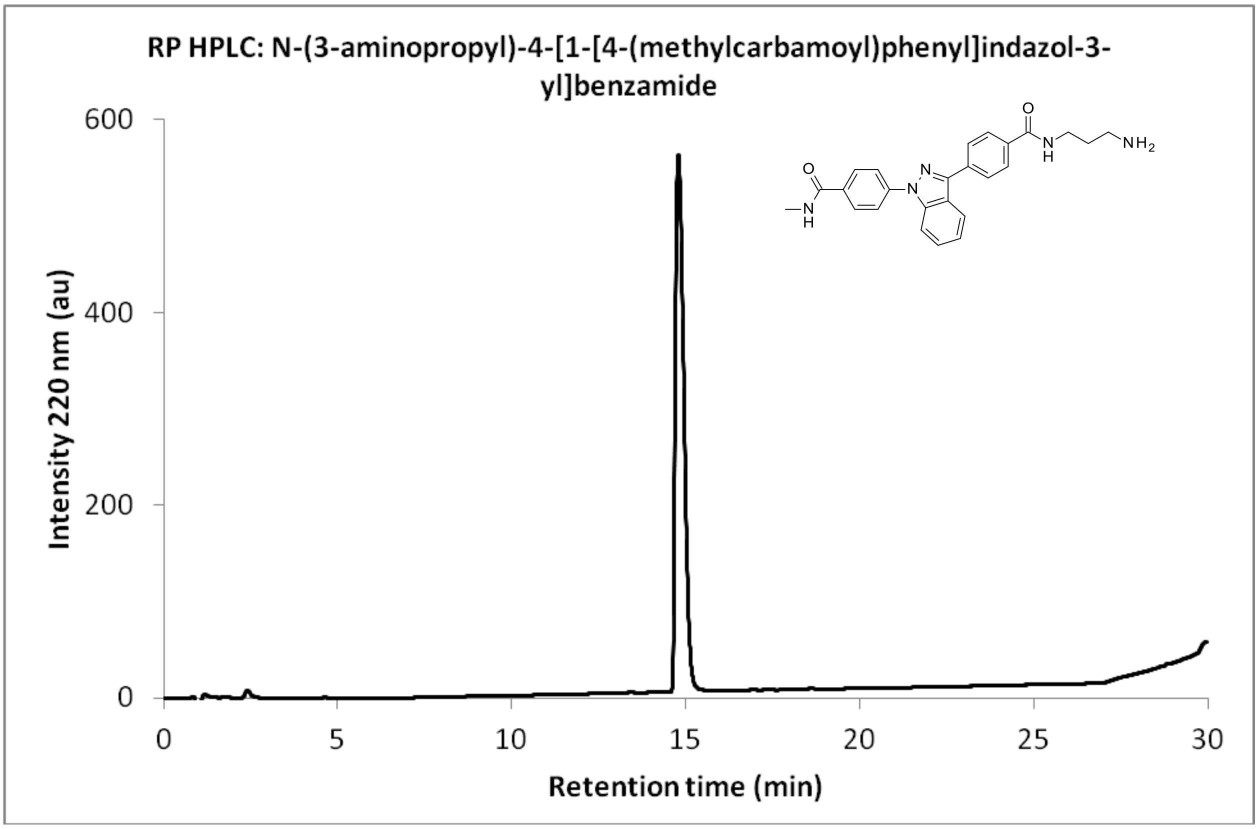
RP HPLC: N-(3-aminopropyl)-4-[1-[4-(methylcarbamoyl)phenyl]indazol-3-yl]benzamide
Intensity 220 nm (au)
Retention time (min)
0
200
400
600
0
5
10
15
20
25
30
O
N
H
NH2
N
N

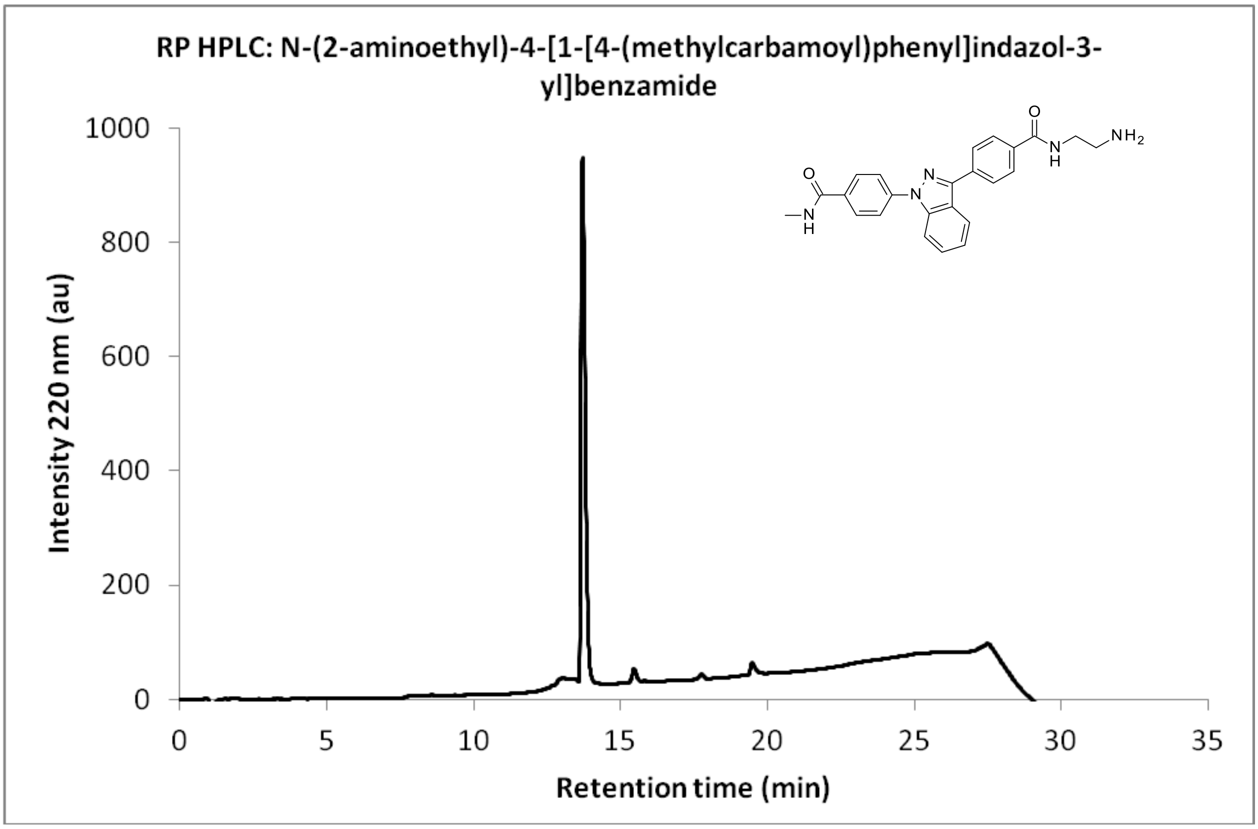
RP HPLC: N-(2-aminoethyl)-4-[1-[4-(methylcarbamoyl)phenyl]indazol-3-yl]benzamide
Intensity 220 nm (au)
Retention time (min)
0
200
400
600
800
1000
0
5
10
15
20
25
30
35
O
N
H
NH2
N
N

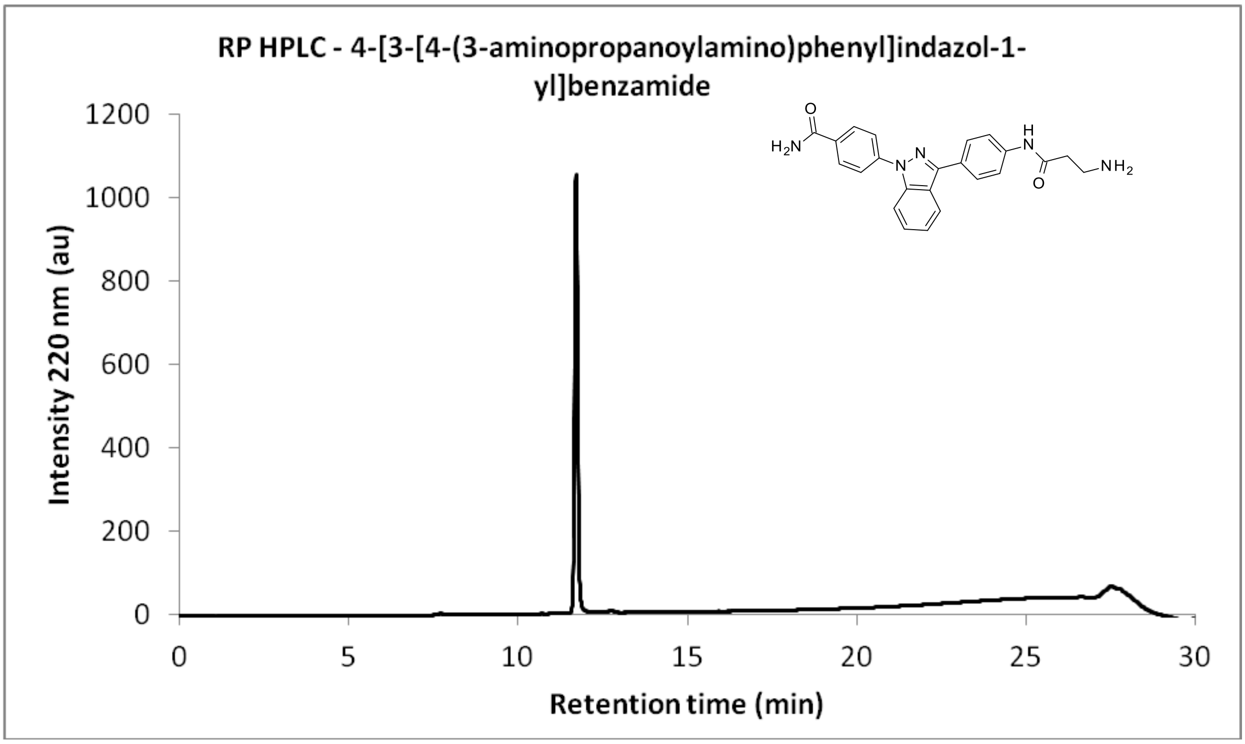
RP HPLC - 4-[3-[4-(3-aminopropanoylamino)phenyl]indazol-1-yl]benzamide
Intensity 220 nm (au)
1200
1000
800
600
400
200
0
0
5
10
15
20
25
30
Retention time (min)
O
H2N
N
N
H
N
O
NH2
